# Second Human Case of Cache Valley Virus Disease

**DOI:** 10.3201/eid1205.051625

**Published:** 2006-05

**Authors:** Grant L. Campbell, James D. Mataczynski, Erik S. Reisdorf, James W. Powell, Denise A. Martin, Amy J. Lambert, Thomas E. Haupt, Jeffrey P. Davis, Robert S. Lanciotti

**Affiliations:** * Centers for Disease Control and Prevention, Fort Collins, Colorado, USA;; † All Saints Healthcare System, Racine, Wisconsin, USA;; ‡ Wisconsin State Laboratory of Hygiene, Madison, Wisconsin, USA;; § Wisconsin Department of Health and Family Services, Madison, Wisconsin, USA

**Keywords:** Cache Valley virus, arbovirus, orthobunyavirus, Bunyamwera serogroup virus, meningitis, encephalitis

## Abstract

We document the second known case of Cache Valley virus disease in a human. Cache Valley virus disease is rarely diagnosed in North America, in part because laboratories rarely test for it. Its true incidence, effect on public health, and full clinical spectrum remain to be determined.

Cache Valley virus (CVV), a mosquitoborne member of the Bunyamwera serogroup, family *Bunyaviridae*, genus *Orthobunyavirus*, is geographically widespread in North America, where it circulates between mosquitoes and mammals (*1*). It has previously been associated with only a single case of human disease, a fatal case of acute encephalitis in the southeastern United States (*2*). We describe the second documented human case of CVV disease.

## Case Report

In late October 2003, a 41-year-old, generally healthy Wisconsin man, who lived near a landfill in the suburbs of a small city on the Lake Michigan shore, became acutely ill with severe headache, nausea, vomiting, and fatigue. The next day, he was hospitalized with a diagnosis of acute aseptic meningitis. On admission, his body temperature was 38.4°C (101.1°F); no neck stiffness, rash, or focal neurologic abnormalities were detected. Computerized tomographic and magnetic resonance imaging scans of the brain were normal. The peripheral leukocyte count was 13,900/mm^3^, with 87% neutrophils and 7% lymphocytes. Cerebrospinal fluid (CSF) examination showed 865 leukocytes/mm^3^, with 73% lymphocytes, 15% monocytes, 12% neutrophils, and no erythrocytes; a protein concentration of 105 mg/dL, (normal 15–45 mg/dL); a glucose concentration of 47 mg/dL (normal 50–80 mg/dL); negative Gram stain; negative latex agglutination test results for antigens of *Neisseria meningitidis* groups A, B, C, Y, and W135, *Haemophilus influenzae* type b, *Streptococcus pneumoniae*, *Escherichia coli* K1, and group B streptococci (Directigen Meningitis Combo Test Kit, BD, Franklin Lakes, NJ, USA); and negative routine bacterial cultures. Empiric intravenous antimicrobial drugs and corticosteroids were begun, and pain medications were administered. After 3 days, the patient's condition improved, and he was discharged on a tapering course of oral corticosteroids. Four months later, he reported feeling fully recovered except for experiencing headaches more frequently than usual.

After the patient's hospital discharge, the Wisconsin State Laboratory of Hygiene isolated a virus (designated strain WI-03BS7669) from an acute-phase CSF specimen. This isolate caused extensive cytopathic effects (CPE) in A549 (human lung adenocarcinoma) cells by 3 days after infection and in RD (human embryonal rhabdomyosarcoma) cells by 6 days, but no CPE were seen in primary monkey kidney or WI-38 (human embryonic lung) cells. Fluorescent-antibody test results of cell culture material were negative for adenoviruses, cytomegalovirus, varicella-zoster virus, herpes simplex virus, and enteroviruses, and polymerase chain reaction (PCR) assays for enteroviruses were negative. When electron microscopy of culture material showed virions morphologically similar to bunyaviruses, the isolate was sent to the Arboviral Diseases Branch of the Centers for Disease Control and Prevention (CDC) for characterization. By using primers targeted to a highly conserved 251-base portion of the smallest of the 3 RNA segments (RNA-S) of members of the Bunyamwera and California serogroups of the family *Bunyaviridae*, strain WI-03BS7669 was shown by PCR to share considerable homology with members of these serogroups (*3*). Subsequent nucleotide sequencing of ≈84% of RNA-S (795 of 950 total nucleotides in genome positions 84–878, GenBank accession no. DQ315775) followed by a BLAST (Basic Local Alignment Search Tool) search in GenBank showed that strain WI-03BS7669 was 99% identical to prototype CVV strain 6V633 (GenBank accession no. X73465; R.M. Elliott, pers. comm.) but only 90% identical to several other Bunyamwera serogroup viruses, including Potosi, Northway, Maguari, and Bunyamwera (*4*). In addition, a 694-base fragment amplified from the RNA-M segment of WI-03BS7669, followed by nucleic acid sequence analysis and a BLAST search, showed 98% sequence identity with 6 CVV strains but only 77% identity with Maguari virus (*4*).

No acute-phase serum was available for arboviral serologic testing. However, convalescent-phase serum collected from the patient 4 months after illness onset was strongly positive (titer 1,280) for neutralizing antibody to CVV strain 6V633 in 90% plaque-reduction tests conducted at CDC.

## Conclusions

The Bunyamwera serogroup includes ≈30 viruses, with known representatives on every continent except Antarctica (R. Weir, pers. comm.) (*1**,**5**,**6*). Transmission cycles for these viruses have been little studied, but most isolates have been from culicine and anopheline mosquitoes, and mammals are considered the primary amplifying hosts. In Africa and Central and South America, Bunyamwera serogroup viruses cause sporadic human illness, including undifferentiated febrile illness, fever with exanthem, meningitis, and rarely, encephalitis. At least 7 different Bunyamwera serogroup viruses have been isolated from humans, usually from blood, including Bunyamwera, Germiston, Ilesha, and Shokwe viruses in Africa; Xingu virus in South America; Wyeomyia virus and a Cache Valley–like virus in Central America; and CVV in the southeastern United States (*1**,**2**,**5**,**7**,**8*). Ngari virus, a reassortant bunyavirus with RNA segments from both a Bunyamwera serogroup virus and an unidentified member of the genus *Orthobunyavirus*, has recently been associated with epidemic hemorrhagic fever in Africa (*9*).

CVV is 1 of at least 9 Bunyamwera serogroup viruses in North America (*1**,**5**,**6**,**10*), where it was first isolated from *Culiseta inornata* mosquitoes collected in Cache Valley, Utah, in 1956 (*5*). Although it has been isolated from >20 different species of culicine or anopheline mosquitoes, most frequently from *Anopheles quadrimaculatus* (*1**,**5*), the principal mosquito vectors are unknown. The vertebrate amplifying hosts of CVV have been little studied, but a high prevalance of neutralizing antibody to this virus is often found in ungulates, including deer, sheep, horses, and cattle (*5**,**10**–**12*). The virus has been isolated from a healthy cow and a sick sheep in Texas (*13*) and from a healthy horse in Michigan (*12*). It is teratogenic in sheep (*14*).

Previous serosurveys have indicated that humans in some parts of the United States are commonly infected by Bunyamwera serogroup viruses. For example, neutralizing antibody to CVV was found in 12% of 356 persons surveyed in Maryland and Virginia in the 1960s (*15*). Such results, however, are often difficult to interpret because of nonrandom sampling, multiple Bunyamwera serogroup members circulating in the same area, inclusion of a limited number of viruses in tests, and serologic cross-reactivity among members of the serogroup.

Only 2 cases of human disease due to Bunyamwera serogroup viruses were previously reported in temperate North America. The first was an encephalitis case in Indiana attributed to Tensaw virus in 1964 (*5*). Unfortunately, because no details about this case or the method of diagnosis were provided, and because the known range of Tensaw virus does not include Indiana, the validity of this report is uncertain. The second was a fatal, culture-confirmed case of CVV encephalitis in a young adult in North Carolina in 1995 (*2*).

Thus, our case of CVV meningitis is only the second documented human case of CVV disease. This case apparently lacked any unique clinical or routine laboratory features, and the diagnosis of CVV disease was made by the isolation of this virus from CSF by a state public health reference laboratory. The viral genomic sequence data and high-titer neutralizing antibody to CVV in the patient's convalescent-phase serum confirmed this case to be an acute CVV infection. Few CSF specimens are cultured for arboviruses because relatively few diagnostic laboratories have the expertise to do so and because even in acute, serologically confirmed cases of neuroinvasive arboviral disease, the isolation rate from CSF is generally low. No tests for CVV immunoglobulin M, such as enzyme immunoassay, are available. Tests for neutralizing antibody to this virus require handling live virus under biosafety level 2 containment and thus are only available by special request at CDC (through state health departments) and selected reference laboratories. In conclusion, CVV disease is a neuroinvasive illness rarely diagnosed in North America, in part because laboratories rarely test for it. Its true incidence, effect on public health, and full clinical spectrum remain to be determined.
